# Editorial: Endogenous danger signals in cancer immunology and immunotherapy

**DOI:** 10.3389/fimmu.2024.1438972

**Published:** 2024-06-13

**Authors:** Xiubao Ren, Jinming Gao, Jingting Jiang

**Affiliations:** ^1^ Tianjin Medical University Cancer Institute & Hospital, National Clinical Research Center for Cancer, Tianjin’s Clinical Research Center for Cancer, Key Laboratory of Cancer Immunology and Biotherapy, Tianjin, China; ^2^ Department of Cell Biology, University of Texas Southwestern Medical Center, Dallas, TX, United States; ^3^ Department of Tumor Biological Treatment, First People's Hospital of Changzhou, Changzhou, China

**Keywords:** endogenous danger signals, DAMP, alarmin, cancer, immunology, immunotherapy

## Introduction

Endogenous danger signals, also known as damage-associated molecular patterns (DAMPs) or alarmin, play a pivotal role in bridging innate and adaptive immunity, particularly in the context of cancer. These molecules, released following cell stress, damage, or non-physiological cell death, activate immune responses and influence cancer immunotherapy outcomes. In this editorial, we explore the multifaceted role of endogenous danger signals, highlighting their impact on antigen-specific immune responses and their complex involvement in cancer immunity.

Despite the remarkable advancements in cancer immunotherapy that have revolutionized cancer treatment, a profound understanding of the intricate immune response mechanisms remains paramount. Endogenous danger signals have emerged as pivotal factors in this domain, encompassing molecules such as defensins, heat shock proteins, cytokines, and others. These molecules function as crucial immune messengers, intricately shaping the tumor microenvironment and ultimately steering therapeutic outcomes.

In the Research Topic “Endogenous Danger Signals in Cancer Immunology and Immunotherapy”, six articles have been published covering various aspects, from systematic reviews of endogenous danger signals to summary of their roles in specific tumor types, from the revelation of new functions of well-known endogenous danger signal to new attempt to apply endogenous danger signals in the cancer immunotherapy. This Research Topic showcased a small but significant portion of the multifaceted functions of endogenous danger signals in cancer immunity.

## Highlights from contributions of this research topic

In this Research Topic, three detailed reviews depicted a big picture of the role of endogenous danger signals in cancer immunity and immunotherapy. The review by He et al. explained the impact of different types of cancer cell death on the immune response against cancer, and introduced several endogenous danger signals, such as the well-known alarm HMGB1. The review contributed by Yang et al. unfolded the controversial role of interleukin-1 (IL-1) family alarmin IL-33 in lung cancer. As a dual functional alarmin with both cancer promoting and inhibiting potential, IL-33 served as a typical example for us to understand the multifaceted effects of endogenous danger signals in cancer. On the other hand, the review by Wu et al. elaborated in detail the contribution of different endogenous danger signals, including IL-33, HMGB1, defensins, and cathelicidins, to the gastric mucosal barrier and summarized their role in gastric mucosal diseases. This is an excellent example for us to understand the complex role of multiple endogenous danger signals within same disease.

The research paper contributed by Selnø et al. and Pirani et al. both focused on the role of the famous alarm HMGB1 in promoting cancer progression. HMGB1 has originally been demonstrated to promote Th1 immune responses involved in protective antitumor immunity. However, soon after, HMGB1 has also been demonstrated to enhance immune suppression by facilitating the differentiation and suppressive activity of myeloid-derived suppressor cells, as well as participating in cancer cell migration and metastasis, and therefore posed prognostic significance for several types of cancer. The multiple roles of HMGB1 in cancer immunity remain to be elucidated. Selnø et al. reported that HMGB1 can induce production of TGF-β via TLR4 in TLR4-positive cancer cells or myeloid cells, and TGF-β can in turn induce production of galectin-9, which is an immunosuppressive protein, in cancer cells. By employing a newly established mass spectrometry protocol, Pirani et al. revealed that cancer cells with high metastatic potential can maintain HMGB1 in a reduced form through thioredoxin system, and the reduced HMGB1 can bind to chemokine CXCL12 and enhance the chemotactic activity of CXCL12, resulting in directional migration and invasiveness of these cancer cells. These two research papers further deepen our understanding of the role of HMGB1 in cancer immunity.

The research paper contributed by Li et al. is a new attempt to apply endogenous danger signals in cancer immunotherapy. They demonstrated that LTX-315, a synthetic cationic oncolytic peptide, induces immunogenic cell death and triggers the maturation of DCs through the release of DC-maturing DAMP/alarmin from dying cancer cells. This work supports the hypothesis that oncolytic and immunostimulating therapeutics can mutually promote the development of antitumor immunity.

## Conclusion and prospection

There are currently several research directions in the field of endogenous danger signals in cancer immunity:

1. Identification of novel endogenous danger signals:
  ◦ Explore additional molecules involved in cancer immunity beyond the well-established DAMPs.
  ◦ Investigate their cellular sources, release patterns, and functional relevance.
2. Mechanisms underlying endogenous danger signal-mediated immune responses:
  ◦ Unravel the intricate signaling pathways triggered by endogenous danger signals.
  ◦ Understand how these signals modulate immune cell recruitment, activation, differentiation, and function.
3. Exploring the role of endogenous danger signals as biomarkers
  ◦ Examine the roles of endogenous danger signals as diagnostic biomarkers, prognostic biomarkers, as well as biomarkers of immunotherapy efficacy.
  ◦ Consider their impact during different stages of disease progression.
4. Translational research of endogenous danger signals in cancer immunotherapy
  ◦ Exploring the potential use of endogenous danger signals, as adjuvants, immune stimulators, synergistic agents for chemotherapy and radiotherapy, or even as therapeutic targets, for cancer immunotherapy.
  ◦ Clinical trials evaluating the efficacy of endogenous danger signal-based therapies, especially in combination immunotherapy.

As we further understand the complexity of endogenous danger signals, we acquire profound insights that have the potential to guide the development of precision cancer immunotherapies. This Research Topic holds significant potential to serve as a pivotal platform for fostering interdisciplinary cooperation, thereby advancing our comprehension of this field, and ultimately unveiling a promising trajectory for the development of innovative combination immunotherapy strategies for cancer.

## Author contributions

XR: Writing – original draft, Writing – review & editing. JG: Writing – original draft, Writing – review & editing. JJ: Writing – original draft, Writing – review & editing.

